# Research integrity in practice: Qualitative analysis of team members’ reflection reports highlights the importance of ongoing collaborative learning

**DOI:** 10.12688/openreseurope.23645.1

**Published:** 2026-07-08

**Authors:** Natalie Evans, Jenny T. van der Steen

**Affiliations:** 1 Department of Ethics, Law and Humanities, Amsterdam Public Health Institute, Amsterdam UMC Locatie VUmc, Amsterdam, North Holland, 1081 HV, The Netherlands; 2 Department of Public Health and Primary Care, Leiden University Medical Center, Leiden, South Holland, 2333 ZA, The Netherlands; 3 Department of Primary and Community Care and Radboudumc Alzheimer Center, Radboud University Medical Center, Nijmegen, 6525 GA, The Netherlands; 4 Cicely Saunders Institute of Palliative Care Policy & Rehabilitation, London, England, UK

**Keywords:** Research Integrity, Responsible Conduct of Research, Research practice, Research Activity, Palliative Care

## Abstract

**Background:**

Universities have a responsibility to provide research integrity (RI) training. Moreover, there is growing recognition that RI training should be continuous and the content discipline- and domain-relevant. In this study, we explore the experiences of researchers of a RI initiative embedded within a domain-specific programme, namely ‘end-of-life’ research. End-of-life research represents a potentially ‘high-risk’ domain when it comes to RI, due to the substantial methodological and ethical challenges involved in researching this patient population, and it can provide an exemplary case for domain specific initiatives. Continuous attention to RI was embedded in The CONT-END end-of-life research programme. The programme’s RI component consisted of regular team meetings to discuss hypothetical RI dilemmas; a two-day RI course; and collaboration with a researcher conducting a meta-research study on end-of-life research.

**Methods:**

Participating researchers’ experiences and perceptions of the RI component were collected through reflective essays half-way through the project (2022/23). Two researchers conducted a reflexive thematic analysis of eight reflective essays from PhD candidates, postdoctoral fellows and an intern.

**Results:**

Three main themes, each with two sub-themes, were developed from the collaborative analysis. These were: (1) ‘Learning’ integrity (subthemes: Collaborative team learning from cases, and Learning principles and rules); (2) ‘Being’ a good researcher and ‘doing’ good research (subthemes:‘Being’ more aware of RI, and ‘Doing’ better research); (3) ‘Influencing’ and ‘being influenced’ by the team (subthemes: Normalising and giving space to RI, and The influence of, and on, team dynamics including team building).

**Conclusion:**

This qualitative evaluation of a multi-layered, continuous RI initiative situated within a domain-specific research area showed the value of case-based collaborative learning both for learning about RI and for team building and socialization as a researcher. Future research should assess what types of training, including collaborative learning initiatives, have impact on how research is actually being conducted in research practice.

## Introduction

Researchers in the field of research integrity (RI) and meta-research are part of a select community with heightened interest in, and awareness of, the trustworthiness of research processes and results. Research integrity refers to “the attitude and habit of the researchers to conduct their research according to appropriate ethical, legal and professional frameworks, obligations and standards. It describes an approach for conducting and organising good scientific work” ( ENERI 2019), whereas meta-research encompasses all “research on research”, including its “methods, reporting, reproducibility, evaluation and incentives” ( Ioannidis 2018). Outside of the ‘RI’ and ‘meta-research’ bubbles, however, most researchers have limited engagement with RI concepts and debates beyond, perhaps, a one-off RI course during a PhD trajectory ( Abdi et al., 2021). There are however institutional obligations to provide training in RI for all researchers ( ALLEA 2023) and, going even further, there have been numerous calls for a deeper engagement of all researchers in continuous reflection and discussion on the means, goals and practice of their profession ( Macfarlane 2010, Mustajoki and Mustajoki 2017, Labib et al., 2022), with a particular need for discipline or domain specific initiatives ( Pizzolato and Dierickx 2021, Labib et al. 2022).

End-of-life research represents one domain, and arguably a “high-risk” one, where attention to RI is particularly warranted. ‘End-of-life’ research aims to develop the knowledge base related to “dying persons, their families, and end-of-life care” ( George 2002). High-quality end-of-life care is important due to the high and enduring impact of the last phase of life, which continues to shape the life of the bereaved. This field faces distinct methodological and ethical challenges ( Aktas & Walsh 2011; Vlckova et al. 2024): conceptual unclarity related to what constitutes the ‘end-of-life’ phase ( George 2002, Browne et al. 2021), the diversity and vulnerability of the ‘end-of-life’ population ( van Mechelen et al. 2012), reliance on proxies to report on end-of-life experiences ( George 2002), and the frequent use of small convenience samples ( Grande & Todd, 2000). Randomized controlled trials, which can be unethical or unfeasible in end-of-life care settings, are rare, meaning researchers rely on more flexible designs ( Grande & Todd, 2000; Gysels et al. 2013; Voznyuk et al., 2025) derived from a variety of disciplines ( Deliens, 2024).

End-of-life care researchers also often come to the area of study due to a deep commitment to improving care for people at the end of their lives. Whilst this is admirable, it is also a challenge if a desire to make palliative care “work” or fixed beliefs of what is good for a patient influence the conduct of research and the interpretation of results ( Randall & Downie, 2006). Indeed, motivations (e.g. a preference for particular findings) and means (e.g. a flexible study design) have been conceptualized as two necessary conditions of reporting bias ( van der Steen et al., 2019). There may also be pressure to demonstrate positive output from limited funds. The RI challenges related to conducting research on the end-of-life population deserve domain specific initiatives, however such domain specific initiatives are scarce.

This paper presents a qualitative evaluation of a RI initiative embedded within an end-of-life research programme, the ‘CONT-END’ programme (“Attempts to Control the End of Life in People with Dementia: Two-level Approach to Examine Controversies”). The CONT-END programme combined clinical research on dementia and end-of-life care, meta-research examining the conduct of clinical research on end-of-life care, and RI education for researchers across the whole project. End-of-life researchers in the programme were offered regular team meetings to discuss hypothetical RI dilemmas, a two-day RI course, and close collaboration with a PhD student conducting a meta-research study on RI in end-of-life research.

The integration of RI research and training within CONT-END provided contextualised and continuous attention to integrity, implemented within an end-of-life research group. While the intervention was continuous over a long period, and involved diverse activities, it involved a relatively small team of researchers. A quantitative evaluation of outcomes was therefore not feasible. Instead, a qualitative approach was more suitable to explore participants’ understanding of course content, engagement in learning activities, and reflection on their own practices ( Tammeleht and Löfström, 2025). Reflexive essays and course work, such as case analyses, which are sometimes used to assess individual learner’s progress in ethics courses (individual level outcomes), can also be valuable for assessing interventions more broadly (group level outcomes). A number of RI and ethics initiatives have evaluated group level outcomes through analysis of course work or reflexive essays ( Tammeleht et al., 2019; Bagdasarov et al., 2012; van den Hoven et al., 2023). These studies explored diverse impacts of initiatives and found, variously, that RI and ethics courses improved conceptual understanding ( Tammeleht et al., 2019), sensemaking and ethical decision-making ( Bagdasarov et al., 2012), and feelings of empowerment ( van den Hoven et al., 2023). To date, however, little empirical work has examined how integrity initiatives can be embedded within ongoing domain-specific research programmes, particularly in fields as ethically and methodologically complex as end-of-life care. Such integrated initiatives can also prove exemplary for other, particularly ‘high risk’ research domains.

The aim of this study is therefore to explore researchers’ perceptions of the benefits of continuous, embedded attention to RI, situated within a high-risk research context, as expressed in reflective essays.

## Method

### Study design

This study employed a qualitative design to examine end-of-life researchers’ perceptions of the benefits of a RI initiative embedded within the CONT-END programme, a six and a half -year project (December 2018–June 2025) on dementia and end-of-life care. Data were collected through reflective essays written by CONT-END programme participants half-way through the project. A reflexive thematic analysis approach ( Braun & Clarke 2006; Braun & Clarke 2019) was adopted, recognising the constructivist and interpretive nature of the evaluation.

### Intervention description

The RI and meta-research component of the CONT-END programme consisted of three aspects:

1. *Regular team meetings to discuss hypothetical RI dilemmas*, attended by the whole CONT-END team consisting of senior researchers, post-doctoral researchers, PhD students and interns. Discussions were centered around the dilemmas from the Erasmus Rotterdam Dilemma Game ( Erasmus University Rotterdam, 2020). At the end of the meeting, a team member volunteered to select the dilemma for the next meeting. Meetings were between 0.5–1.0 hour long (longer sessions included a round on how the researchers were doing more generally), and occurred from the second year every two weeks for two years, and every month for the remainder of the project;
2. *A two-day RI course*, for the team’s PhD students and postdoctoral fellows. Although this was supposed to be an in-person course, it was moved online due to the Covid pandemic. Also due to staff turn over, 4 included participants did not attend the course;
3. *Meta-research component within the broader project.* The novel CONT-END programme consisted of three work packages (WPs): two on dementia and the end of life, and a third meta-research WP on RI in end-of-life research. One of the team members was a researcher fully employed on the meta-research study.

### Participants recruitment

All end-of-life researchers affiliated with the CONT-END programme in 2022 or 2023 (at the programme midpoint) were invited to participate. Eligible participants were PhD candidates or postdoctoral researchers who (a) worked on a dementia and end-of-life WP of the CONT-END programme or a related dementia care project under supervision of a CONT-END team member, (b) had been part of the research team for at least six months, (c) participated in regular team meetings, and (d) were not directly involved in the meta-research component of the initiative, as these researchers were primarily conducting RI research rather than end-of-life research.

Of the nine eligible researchers working on CONT-END midway of the programme, all submitted reflective essays. Following re-consent procedures for blinded analysis and publication, one participant withdrew their essay. The final dataset therefore comprised eight reflective essays.

### Data collection

The participants were asked to write a reflective essay (in English or Dutch) on their experience of the multi-layered, continuous RI WP (consisting of the three aspects listed above), to answer the question: “What did you, as a CONT-END team member and researcher on end of life with dementia, experience from the CONT-END project’s RI WP, and was this of any benefit? Please reflect.” No minimum or maximum word count was initially given for the essays, but in follow-up requests, half to 2 A4 pages was suggested as a guideline based on what had been submitted up to that point. Submissions ranged from 222 to 889 words, however the mean length was 427 words. These were very rich texts as they were aimed at directly answering the research questions.

### Ethics and informed consent

**Ethics approval:** The RI component of the CONT-END programme was declared exempt from the Medical Research Involving Human Subjects Act (WMO) at 23 September 2020 by the Medical Research Ethics Committee Leiden Den Haag Delft (reference N20.131).

When invited to voluntarily write their essays, participants were informed that their reflections would be used to evaluate the CONT-END programme’s embedded RI initiative. All participants gave written consent for initial analyses. The essays were not used for any other purpose. They were not part of the formative or summative assessment of any course. At a later stage, NE was invited to independently analyse the essays; participants were re-contacted and asked to provide additional consent for a second analyst to be involved. One participant declined, resulting in the exclusion of their essay from analysis.

### Positionality statements

JvdS is the PI of the CONT-END programme and has extensive experience in dementia and end-of-life research. She has also done some research on RI. Her mission is to employ thorough methodology and adhere to standards of RI to perform high-quality research on end-of-life with dementia. JvdS involved NE because of her previous research in palliative care, and current research and teaching on RI and skill in qualitative work, and as a counter-balance for JvdS’s personal conviction about the value of the RI WP.

NE is an experienced qualitative researcher in both RI and end-of-life care. NE has focused her research in recent years on RI education. NE was not involved in the CONT-END programme but was invited to independently analyse the reflective essays. At the time of conducting the independent analysis, NE was the coordinator of the same formal RI course that the participants undertook in 2020. She was not coordinating or teaching when the CONT-END researchers (who worked at a different university) undertook the course. She does, however, have a thorough understanding of the content of the course at that time.

The researchers’ backgrounds overlapped yet brought distinct professional and experiential lenses to the analysis, and their distinct perspectives were treated as a resource for critical reflexive dialogue.

### Data analysis

Personal identifying information was removed from the reflective essays, which were subsequently line by line inductively coded by JvdS and NE. Both researchers developed coding trees and even preliminary themes independently after coding all eight essays in MAXQDA (VERBI Software, 2021). These independent analyses were presented as posters at the 8 ^th^ World Conference Research Integrity in Athens in 2024 ( https://zenodo.org/records/19483351) to spark discussion on the constructivist nature of qualitative evaluations.

JvdS’s initial codes were primarily oriented toward issues of team climate, communication, and roles, while NE’s analysis emphasised learning processes and typologies. However, NE also included codes for power dynamics and hierarchies, and JvdS included codes on collaborative learning. These differences were used as a source of insight for a series of reflexive discussions on the initial codes and preliminary themes and how these were influenced by each researcher’s relationship with the context and the research subject. The two researchers subsequently used the preliminary coding schemes to develop a single coding scheme reflective of a joint interpretation of the data arrived at through iterative discussion and comparison. This final coding tree was used to code all essays. A comparative table of segments coded by each researcher for each code was produced in MAXQDA and discrepancies were discussed in a series of online and face-to-face sessions– leading to refinements to code descriptions and greater consistency of application of the coding tree. Final codes were then organized into themes and sub themes which best fitted the joint interpretative understanding of the underlying data in collaborative sessions between NE and JvdS. .

## Results

Of the 9 team members who had written reflexive essays, 6 PhD students and 2 postdoctoral fellows also consented to independent qualitative analysis by NE. The final codes and developed themes are provided in Table 1. Three main themes, each with two sub-themes, were developed from the collaborative coding process. These included (1) ‘Learning’ integrity (subthemes: Collaborative team learning from cases, and Learning principles and rules); (2) ‘Being’ a good researcher and ‘doing’ good research (subthemes:‘Being’ more aware of RI, and ‘Doing’ better research); (3) ‘Influencing’ and ‘being influenced’ by the team (subthemes: Normalising and giving space to RI, and The influence of, and on, team dynamics). These themes reflect the main insights from the qualitative evaluation of the RI initiative and are described below with illustrative quotes from participants’ reflective essays.

**Table 1.** Final themes, subthemes and codes of experiences of end-of-life in dementia researchers in a programme with an embedded RI WP.

Theme	Subtheme	Code	Code description
**1. ‘Learning’ integrity**	**1.1 Collaborative team learning from cases**	***a Learning from simulated cases***	Coded here: use and experience of hypothetical cases to stimulate discussions.
		***b Awareness and knowledge of grey areas***	Coded here: knowledge and awareness everyday dilemmas. Opposite also coded here: idea that there are easy/perfect solutions.
		***c Team based learning***	Coded here: descriptions of team-based learning approaches; group learning processes; coming to group consensus; co-creation. Not coded here: quality of team interactions - this is coded under Team dynamics (3.2a). Not coded: discussion of RI in daily practice - coded with Normalized practice (3.1a).
		***d Learning from diverse perspectives***	Coded here: different angles, perspectives, knowledge. Not coded here: diversity in team background - coded under learning from diverse team backgrounds. Not coded here: learning from different experiences - coded under learning from shared experiences.
		***e Learning from diversity in team background***	Coded here: diversity in team background, in terms of discipline and seniority. Not coded here: different angles, perspectives, knowledge - coded under diverse perspectives and backgrounds. Not coded here: learning from different experiences - coded under learning from shared experiences.
		***f Learning from direct experience***	Coded here: learning via experiencing first-hand a questionable or good research practices (QRPs or GRPs); learning from role models (good and bad).
		***g Learning from sharing personal experiences***	Coded here: learning via hearing about the first hand experiences of other related to QRPs or GRPs.
		***h Awareness of the academic world outside the team***	Coded here: influences outside team or influence of culture of science; other influences on research careers (precarious careers).
	**1.2 Learning principles and rules**	***a (not via) Theoretical approach of strict boundaries?***	Coded here: Typical RI content (falsification, fabrication and plagiarism); principles; guidelines.
		***b Learning from a formal course***	Coded here: experience of a formal course, interaction in the course, delivery of the course, perspectives on the course.
**2. ‘Being’ a good researcher and ‘doing’ good research**	**2.1’Being’ more aware of RI**	***a Recognising and navigating ethical issues***	Coded here: recognising and approaching ethical dilemmas/decisions in research (i.e. interpersonal issues).
		***b Prevention of behaviour***	Coded here: awareness preventing QRPs; QRPs difficult to avoid in practice.
		***c Intentions re. behaviour***	Coded here: descriptions of what participants hope to do in the future re. QRPs/GRPs Not coded here: future role of supervisor - coded in “As a (future) supervisor (2.2c)”.
		***d Better person***	Coded here: acknowledge vulnerabilities; increase moral sensitivity.
	**2.2 ‘Doing’ better research**	***a Actual behaviour***	Coded here: actual descriptions of behaviour change in practice.
		***b Navigating methodological/professional issues***	Coded here: methodological competencies; other professional competencies.
		***c As a (future) supervisor***	Coded here: Intentions related to practice as a supervisor.
**3.‘Influencing’ and ‘being influenced’ by the team**	**3.1 Normalising and giving space to RI**	***a Normalized practice***	Coded here: discussion of RI within the team; frequent attention to RI; RI systematically addressed/reflected on. Not coded here: discussion of RI in context of course with participants other than team.
		***b When Just given its space***	Coded here: giving attention and space to RI, RI valued.
	**3.2 The influence of, and on, team dynamics**	***a Team dynamics***	Coded here: quality of interactions between team members generally. Not coded here: interactions about research.
		***b Sense of hierarchy***	Coded here: opinions about the need for/impact of RI initiatives among different career stages; experiences of power differences; awareness of position in team.
		***c Through team building***	Coded here: team building/bonding via RI activities.
		***d Via sense of harmony (open, safe, enough)***	Coded here: descriptions of team being open, a safe space, one in which a team member feels confident enough; also coded the opposite - not safe, not open.

### Theme 1: ‘Learning’ integrity

***1.1 Collaborative team learning from cases***

Participants wrote most frequently, and most positively, on learning about the ‘grey’, ‘nuance’, and ‘complexity’ of real life RI issues, particularly in relation to participation in team meetings to discuss hypothetical RI dilemmas through the use of the Erasmus Rotterdam Dilemma Game.

*“These discussions have played a pivotal role in drawing my attention to nuanced ethical considerations that may arise during my research career.” **PhD student 6.***

These insights were described as having been achieved through discussion with other members of the team. Through sharing perspectives and experiences, participants described coming to view a problem from different perspectives, developing an understanding of diverse influences on decision-making, different ways to approach a problem, and even practicing difficult conversations. These discussions generated new knowledge that could not have been achieved alone: this knowledge was not available in the Dilemma Game hypothetical cases and associated options for action, but rather was achieved through discussion with others.

*“This has exposed me to a diverse range of experiences and ethical viewpoints of the other team members, which has provided me with a broader lens through which I view ethics in research. It has emphasized the nuances and complexities that I may not have initially grasped solely from my own perspective or experiences.”* ***PhD student 6***

*“The discussions at the dilemma game made me more aware of the different factors that may influence critical decisions in research, such as limitation in time and resources and interpersonal relationships among researchers.”* ***PhD Student 2***

*
“[t]he Dilemma Game offered a way to “practice” conversations about difficult decision-making.* ***PhD student 1***

One PhD student described team discussions leading to a consensus about the best response to a situation ( **PhD student 1**), whereas another emphasized that consensus was not the goal, but rather ‘ *to share different perspectives on what constitutes as ethical conduct*’ ( **PhD student 6**). Indeed, the value of learning from the diverse perspectives and experiences of the group was frequently described. Learning from different perspectives was often explicitly linked to diversity within the group in a number of reflections, particularly the mix of people from different career stages.

*“Early career researchers thus get a chance to hear from more senior researchers that science integrity is not always easy to practice, and that we recognize the problems we face in the sometimes toxic academia.” **Postdoc 2***

The value of learning from different perspectives was also reflected on in relation to the disciplinary mix of the team as a whole, beyond the Dilemma Game.

*“What I do find of added value is that because the research integrity WP has a different subject than the other packages, ACP [Advance Care Planning] in dementia, is that you have a PhD in the broader team who has a slightly different background and sometimes looks at certain things in a different way. The type of research that this PhD does is also different from what we do (a lot of qualitative). This brings diversity to your research group”.* ***PhD student 4***

Participants frequently wrote about how the use of hypothetical scenarios in the Dilemma Game facilitated the sharing of perspectives, opinions and experiences, and enabled a feeling of ‘safety’ in discussions.

*“[d]ilemmas related to power issues between a junior researcher and a supervisor could be discussed in a group where both senior and junior researchers were present. Both viewpoints were addressed without it negatively impacting on the relationship between the junior and senior researchers, as the situation did not pertain to an actual situation.”* ***PhD student 1***

These discussions provided an opportunity for juniors to learn, not just about RI, but also about how to be a researcher.

*“Lastly, besides its educational value, the dilemma game is a refreshing experience for me and it is a bonding activity for the team. Talking with colleagues about research itself is fruitful and different from daily project updates and small-talks.”* ***PhD student 2***

The recognition of RI as a complex problem also resonated with participants’ previous experiences in research, which were sometimes referred to in their reflections.

*“As a postdoctoral researcher who has worked at [an anonymized number of] different universities, in [an anonymized number of] different research groups, it is clear to me that scientific integrity is not always black and white, nor easy to implement.”* ***Postdoc 2***

***1.2 Learning RI principles and rules***

Most participants wrote little about learning RI principles and guidelines. When they did, they mostly described principles and rules as abstract or difficult to apply in practice.

*“As a researcher one should always abide to the principles of research integrity: honesty, rigour, transparency and open communication, care and respect and accountability. It seems logical to abide to those rules. However, at the same time these are fairly abstract terms.”* ***PhD student 3***

The Dilemma Game however was described as making these principles ‘less abstract’ by one student ( ***PhD student 3***), whereas another ( ***PhD student 1***) described more junior researchers as learning *‘need to know’* principles important to RI, related to peer review, funding and authorship, through the Dilemma Game discussions.

In contrast, very little was written on the formal taught course, with just three participants mentioning it in their reflections. Those that did were disappointed with a perceived lack of interaction (the course was conducted online during the Covid pandemic) and contrasted the awareness and capacity to engage in nuanced discussion of their team compared with other course attendees. In fact, no participant described having *learned* anything new in the formal course.

*“Unfortunately the course was somewhat disappointing. It included a lot of homework and little interaction … Content-wise, I would have appreciated more focus on smaller breaches of research integrity and the grey area instead of black-white things like data fabrication and falsification.” **PhD student 2***

*“When attending a course on research integrity, it became clear that our team was better able to analyze situations and had a stronger vocabulary to discuss the assignments. While other attendees were more emotional in research integrity discussions or were apprehensive to share their thoughts, our team members were active participants with a more “objective” way of dissecting the dilemma and underlying values”.* ***PhD student 1***

### Theme 2 ‘Being’ a good researcher and ‘doing’ good research

***2.1’Being’ more aware of RI***

Participants frequently stated that their new appreciation of RI would be valuable for them as researchers and for their research practice.

*“I think this diversity of viewpoints and thinking about these different dilemmas has made me a more ethically conscious researcher in general”.* ***PhD Student 6.***

Participants considered the attention to RI as having a preventative function, keeping RI issues at the forefront in decision making in research and in their future role as a supervisor.

*“I would like to think that hearing frequently about the topic of research integrity (by attending team meetings and the Dilemma Game) makes me more alert to those situations”.* ***PhD Student 3***

Indeed, one researcher ( ***Postdoc 2)*** described this ethical awareness as potentially improving overall morality and making researchers “better humans”.

***2.2 ‘Doing’ better research***

One participant concretely described changing their research practice based on their new understanding of the ethical dimensions of research whereas another described becoming more aware of their exemplary role as a supervisor.

*“Openly talking about dilemmas in the team also helped me consider the ethics dimension when making decisions about my own project. For example, we decided to reduce the number of interviews partly in order to reduce data waste and reduce burden to the participants”.* ***PhD student 2***

*“As a supervisor of a PhD candidate, the focus on integrity has made me extra aware of the importance of discussing integrity within research with researchers in training. But also that you have an exemplary function. And therefore also have to conduct research with integrity”(translated from Dutch)* ***Postdoc 3***

Others described the focus on RI as having improved the “quality”, “decision-making” and “methods” of their projects, without providing specific examples.

Although reflections were sometimes started by situating the RI intervention within an end-of-life project, none of the participants reflected on the specific RI challenges related to end-of-life care research itself. One participant did associate the focus on RI in the project with the project subject matter (end-of-life care for people with dementia), however this was framed more broadly as ethically challenging rather than providing RI challenges specifically.

*“The CONT-END Programme focuses on a topic that calls for ethical caution: the end-of-life care in people with dementia. That is why the project lead has chosen to put extra emphasis on ethical aspects of conducting research.”* ***Postdoc 2***

### Theme 3 ‘Influencing’ and ‘being influenced’ by the team

***3.1 Normalising and giving space to RI***

Participants frequently described how just giving RI space in and of itself had an influence on the team. Even when participants described not being directly involved in or having much knowledge of the RI WP, they perceived its inclusion in the larger project as an indication that RI was being valued. This, and regular joint reflection on RI issues, facilitated discussion of, and attention to, RI becoming a ‘normalized’ practice. Most found that the structural implementation of RI discussions helped to keep the topic alive and on the team’s agenda.

“ *In general, feels like this research integrity topic is intertwined in the day-to-day conversations with the CONT-END colleagues*” ***PhD student 5***

***3.2 The influence of, and on, team dynamics***

Participants emphasized however that this normalization requires a safe culture of open dialogue within the team. In general, participants described the RI discussions as a “safe” place. Moreover, the team meetings and discussions were described as having the additional benefit of being “bonding” and “supportive” moments for the team which the participants were “privileged” and “happy and proud” to have participated in.

Open dialogue, however, could be hampered by perceived power imbalances amongst team members. Three participants wrote specifically about how difficult it is to give additional attention to RI, or to question the status quo, due to career stage, job precarity, or cultural differences.

*“In my opinion, early career researchers have to meet impossible standards in an extremely competitive environment, and it is sometimes a challenge to keep that scientific integrity at the forefront of your work … we can be even more reflective by raising more awareness of specific power structures within our research group, or any research group.”* ***Postdoc 2***

Moreover, two participants presented very different perspectives about the power of junior researchers to express themselves freely: one suggested that the ‘flat’ hierarchy in the Netherlands allows junior researchers to express themselves freely in team meetings, unlike in other cultures ( ***PhD student 1***), whereas another described how residency in the country linked to precarious job positions can cause hesitancy to ask critical questions ( ***PhD Student 2***). Highlighting the potential for different team members to have distinct experiences within the same team.

## Discussion

This qualitative evaluation of a multi-layered, continuous RI initiative situated within a specific research area showed that there is value in case-based collaborative learning both for learning about RI and additional influence on team building and socialization as a researcher.

Discussions of hypothetical cases provided a safe environment for participants to bring in and compare their own related experiences from practice, demonstrating the value of both the discussion of the case at hand, and the added learning opportunities related to knowledge collaboratively developed when discussions would branch to personal cases. The value of this shared knowledge would arguably not have been as great if the members of the team discussion had not been diverse in terms of career stage.

Notable in its absence was discussion of learning from role models in practice – indeed the only time role modelling was mentioned was in relation to the participants realizing that they themselves were role models. However, there was more emphasis on the immense value of learning together with researchers of diverse career stages, perspectives and experiences rather than learning from those more experienced alone.

In their reports, the participants hardly reflected on the specific RI challenges of their own domain. Although the knowledge built within the team discussions may be tailored and contextualized to end-of-life research specifically, only the postdoctoral fellows could compare any current experiences with previous experiences with former positions in different domains. Also, the dilemmas (selected by the team) from the Dilemma Game which triggered joint reflection drew on various domains yet were appealing to the team.

The value of case-based collaborative approaches for learning about the responsible conduct of research supports findings from previous studies, such as those conducted among students and early career researchers ( Tammeleht et al., 2019) and postgraduate students and postdoctoral researchers ( McCormack & Garvan 2014). The value of conducting these discussions within research teams among participants of diverse career stages is also clearly demonstrated. Collaborative learning through discussions may also support navigating research decisions which involve uncertainty or moral ambiguity which cannot be resolved by appeal to ethical frameworks and guidelines alone, an observation supported by the findings of the CONT-END programme’s ethnographic fieldwork ( van Drimmelen et al., 2025) and supported by teaching approaches which provide explicit steps for (often collaborative) structured reflection on dilemmas experienced in research ( Shamoo & Resnik 2022; van den Hoven et al., 2023; Inguaggiato et al., 2023; Evans et al., 2023).

The findings of this study have implications for RI teaching. Institutions, at least in Europe, are responsible for supporting RI through the education of students and researchers. Indeed the European Code of Conduct on Research Integrity ( ALLEA 2023) states that training should make researchers ‘aware of the relevant codes and regulations and develop the necessary skills to apply these to their research’ ( ALLEA 2023). To date, most provision is limited to PhD students and many institutions meet provision via online or hybrid courses ( Abdi et al., 2021). This study however indicates that communal reflection, particularly among colleagues at different career stages and with diverse experiences, might be a more valuable approach. Furthermore, the study demonstrates that a continuous attention to RI can be achieved through repeated and relatively short (e.g. just 30 minute) discussions within teams. Replicating a multi-layered RI initiative, such as the RI WP in the CONT-END study, is costly. However the communal discussions were the most valued aspect of the initiative, which is, potentially, more cost effective, frequent and continuous than formal courses.

### Strengths, limitations and future research

The innovative initial independent analysis and theme formation allowed the authors (NE and JvdS) to reflect on their positionality and how their prior experiences and relationship with the intervention influenced their analysis. Joint reflective discussions and analysis allowed the authors to come to a shared understanding and interpretation of the data.

Due to staff turn over, only half of the included participants had undertaken the formal RI course. Ideally, we would support the mixed method evaluation of any RI evaluation, including also quantitative evaluations. The number of end-of-life researchers active midway in the CONT-END programme was too small (just 9) to make that feasible. The sample is also small, and concerns a single research group, however all PhDs and postdocs included in the intervention were invited to reflect on the novel intervention and 8 out of the 9 allowed their reflections to be included. Whilst it is not possible to say if thematic saturation is reached, the study can offer insights which can be further developed in larger interventions in the future.

The participants in our study formed a team with a partly shared understanding of challenges in the specific research domain. Explicit domain-specific teaching rather than implicit learning from those working within a domain might further improve participants understanding of the disciplinary and domain specific challenges they face. Future research should assess what types of training, including collaborative learning initiatives, have impact on how research is actually being conducted in research practice.

## Conclusion

Regular team-based discussions of RI dilemmas enabled the sharing of perspectives and past and present experiences related to RI, and the role of a researcher more broadly. These were highly valued by researchers, who appreciated the influence the discussions had on their understanding, attitudes and intentions. A formal course, in contrast, had little added value because of the considerable learning derived already from regular team-based learning. Interaction between junior and senior researchers within research teams around hypothetical RI issues can offer relatively safe learning experience for juniors to identify, navigate and properly act upon dilemmas encountered in doing research.

## Author roles

Natalie Evans: Methodology; Analysis; Writing – Original Draft; Writing – Review & Editing. Jenny van der Steen: Conceptualisation; Methodology; Investigation; Analysis; Resources; Data curation; Writing – Original Draft; Writing – Review & Editing; Supervision; Funding acquisition.

## Data Availability

The qualitative data are not publicly available in line with data management commitments made to participants during the informed consent procedure, in which participants were informed that the research would be conduct with the intention “to make any reports or publications derived from this study unidentifiable”. Due to the identifiable nature of the small participant group, even pseudonymised data risks recognition.
