# Supplementary material for: Research integrity in practice: Qualitative analysis of team members’ reflection reports highlights the importance of ongoing collaborative learning
Source: Open Res Eur. 2026 Jul 8;6:239. [Version 1] doi: 10.12688/openreseurope.23645.1 (PMC13560594; doi:10.12688/openreseurope.23645.1)
Supplement: Peer review history

# Reviewer report: Reviewer response for version 1

**DOI:** 10.21956/openreseurope.25601.r78271

**Published:** 2026-08-18

**Authors:** Kalle Videnoja

**Affiliations:** 1 The Finnish National Board on Research Integrity TENK, Helsinki, Finland

The manuscript is clear, well structured, and provides a strong and transparent account of the data collection and methodological approach. The analysis is valuable not only for addressing the domain-specific questions, but also for contributing to the wider discussion within research integrity (RI) community of practice.

 The manuscript raises important questions about the sustainability of training initiatives and, more broadly, about the relationship between individual learning and the more collective process of normalising discussion of RI-related issues in researchers’ day-to-day work. 

 A point that could nevertheless be clarified concerns the collection and analysis of the essays. At present, it is somewhat difficult to assess the degree of variation across the essays and whether particular themes and learning outcomes were more pronounced in some essays than in others. Providing clearer information of this variation, and if certain themes were especially prominent across the material, would help the reader better understand the range of the findings.

 The manuscript notes that the participants hardly reflected on the specific RI challenges of their own domain, but it remains somewhat unclear whether the discussions nevertheless revolved around concrete issues that were directly relevant to participants’ research practices, e.g. managing authorship expectations or making data reusable or addressing other practical aspects of responsible research, or whether the discussions tended to remain at a more abstract or general level. 

 There is an observation on the account of PhD student 2, who describes their work as being directly influenced by openly discussing dilemmas within the team. Building on this example, it would be beneficial to distinguish more explicitly between outcomes that remained at the level of discussion, reflection or increased interest in RI, and outcomes with indications of changes in research practices induced by the CONT-END project’s RI WP. 

 fpubli

 A further issue that could be reflected more explicitly is the sustainability of the reported learning outcomes and discussion practices. It would be interesting to know how the role of the Dilemma Game, or other external prompts, is reflected in the essays or interpreted by the authors. Is an external prompt like the Dilemma Game necessary condition for initiating and sustaining an open culture of discussion around RI-related topics or could such discussions become self-sustaining if RI considerations were more systematically incorporated into the day-to-day communication of research teams?

If applicable, is the statistical analysis and its interpretation appropriate?

Not applicable

Is the study design appropriate and is the work technically sound?

Yes

Is the work clearly and accurately presented and does it engage with the current literature?

Yes

Are the conclusions drawn adequately supported by the results?

Partly

Are sufficient details of methods and analysis provided to allow replication by others?

Yes

Are all the source data and materials underlying the results available?

Yes

Reviewer Expertise:

Research integrity, research ethics, training and education, philosophy of law.

I confirm that I have read this submission and believe that I have an appropriate level of expertise to confirm that it is of an acceptable scientific standard.

# Reviewer report: Reviewer response for version 1

**DOI:** 10.21956/openreseurope.25601.r78273

**Published:** 2026-08-14

**Authors:** Lisa DeTora

**Affiliations:** 1 Hofstra University, Hempstead, New York, USA

This is a soundly-conceived and well-executed qualitative research study of researcher experiences with research integrity content embedded in the CONT-END end-of-life research programme.  Doctoral students, posydoctoral fellows and interns were invited to submit reflective essays regarding their experiences, which were coded and used to identify main themes. The researchers found that their limited sample of brief essays revealed three main themes and several sub themes oriented to specific actions.  

 These were ‘Learning’ integrity (subthemes: Collaborative team learning from cases, and Learning principles and rules); ‘Being’ a good researcher and ‘doing’ good research (subthemes:‘Being’ more aware of RI, and ‘Doing’ better research); ‘Influencing’ and ‘being influenced’ by the team (subthemes: Normalising and giving space to RI, and The influence of, and on, team dynamics including team building).

 Overall, the study yielded intriguing insights and the analyses are convincing, especially given the small sample size.  The insights quoted from the research participants highlighted some of the key tensions between mastering new skills and knowledge and maintaining integrity.  There seemed to be a focus on the participants' experience of the programme, with little reflection on patient experiences or benefits.

 The one caveat is that it is unclear how the researchers interpret the fact that this was a study conducted in a specific context (end of life care for patients also suffering from dementia).  The title and the discussion and the analysis could make more specific reference to this setting, especially given the selection of some key quotations from the research participants about their perception that research integrity was especially important in this specific setting.   For this naive reader, it seems important to have the authors' views on that aspect of this study.  

 It might be useful for future studies to ask participants about some moral dimension of this work as regards patient care as well as their educational experience.  A very brief comment on the open-ended nature of the writing prompt and whether more frequent reflections might be beneficial would be helpful to readers who seek to undertake a similar program.

If applicable, is the statistical analysis and its interpretation appropriate?

Not applicable

Is the study design appropriate and is the work technically sound?

Yes

Is the work clearly and accurately presented and does it engage with the current literature?

Yes

Are the conclusions drawn adequately supported by the results?

Partly

Are sufficient details of methods and analysis provided to allow replication by others?

Yes

Are all the source data and materials underlying the results available?

No source data required

Reviewer Expertise:

Bioethics, rhetoric of science, technology, health and medicine, medical humanities, biomedical publication ethics, health communication

I confirm that I have read this submission and believe that I have an appropriate level of expertise to confirm that it is of an acceptable scientific standard, however I have significant reservations, as outlined above.

# Reviewer report: Reviewer response for version 1

**DOI:** 10.21956/openreseurope.25601.r78268

**Published:** 2026-08-14

**Authors:** Seán Lacey

**Affiliations:** 1 Munster Technological University, Rossa Avenue, Ireland

This is a well-written report on the impact of research integrity training within an end-of-life research programme. The manuscript is clearly structured throughout, with sufficient detail in the Methods section to support reproducibility. The findings are presented effectively, the discussion is balanced, limitations are appropriately acknowledged, and the conclusions are supported by the data. I enjoyed reading the manuscript.

 I have three observations for the authors' consideration:

 1. Framing of the study within the abstract and introduction - The opening sentence of the abstract states that *"Universities have a responsibility to provide..."*. This led me to expect a greater degree of framing around the responsibilities of institutions in delivering research integrity training within end-of-life research programmes. While this does not detract from the overall quality of the manuscript, there is relatively little discussion of this aspect in the main text. I am not suggesting a substantial revision, but the authors may wish to either: 

 - reconsider the wording of the opening sentence in the abstract; or

 - provide additional contextualisation in the introduction and/or discussion regarding institutional responsibilities and how the findings may inform organisational practice.

 In addition, the authors may wish to consider using the more inclusive term *Research Performing Organisations (RPOs)* rather than *Universities*, as not all higher education and research institutions operate under a university designation. The findings may be of interest to a broader range of organisations, including institutes of technology and colleges.

 2. Potential identifiability of participants - Participant quotations are attributed to identifiers such as *PhD Student 1* or *Postdoc 2*. Given the relatively small sample size, I wonder whether this level of attribution is necessary. In some local contexts, consistently linking multiple quotations to the same participant category and identifier may increase the risk of participant identification. The authors may wish to consider whether attribution at the level of *PhD participant* or *Postdoctoral researcher* would sufficiently support interpretation without introducing any unnecessary identification risk or simply do not attribute the quotes at all.

 3. Citation of direct quotation - A direct quotation from ALLEA is included in the manuscript. As this is a verbatim quotation, the corresponding page number should be included in the citation.

If applicable, is the statistical analysis and its interpretation appropriate?

Yes

Is the study design appropriate and is the work technically sound?

Yes

Is the work clearly and accurately presented and does it engage with the current literature?

Yes

Are the conclusions drawn adequately supported by the results?

Yes

Are sufficient details of methods and analysis provided to allow replication by others?

Yes

Are all the source data and materials underlying the results available?

Yes

Reviewer Expertise:

Research Integrity; Research Ethics; Open Science; Statistical Analysis; Qualitative Research; Quantitative Reseach.

I confirm that I have read this submission and believe that I have an appropriate level of expertise to confirm that it is of an acceptable scientific standard, however I have significant reservations, as outlined above.

# Reviewer report: Reviewer response for version 1

**DOI:** 10.21956/openreseurope.25601.r78272

**Published:** 2026-08-13

**Authors:** Jacopo Ambrosj

**Affiliations:** 1 Laboratoire Interdisciplinaire Sciences Innovations Sociétés, Marne-la-Vallée, France

**Summary and overall judgement**

 The authors of this study explore the perspectives of researchers continuously exposed to research integrity (RI) initiatives as part of a dedicated work package within a research project on end-of-life care. They do so by developing a reflexive thematic analysis (TA) of 8 essays written by early-career members of the project reflecting on their experience with the RI work package. This included a two-day RI course, regular discussion with team members on RI dilemmas, and close collaboration with a team member working full time on the RI component.

 The research aim is well articulated, well situated within the literature on empirical research on RI, and the chosen method is appropriate and partially original (including two coders within the framework of reflexive TA). Particularly commendable is the authors’ clear description of the research context and reflection on their role and positionality. More engagement with the theoretical literature on RI and further clarification of the more original aspects of the authors’ reflexive TA will further improve the article both conceptually and methodologically. Below, I articulate these two points by expanding on my answers to the questions provided in the peer review form. Finally, I add one very minor point that the authors might feel free to adopt or ignore.

***Is the work clearly and accurately presented and does it engage with the current literature?***

 Partially. The authors engage with other empirical studies on RI educational initiatives but fail to engage with more philosophical reflections on RI.

 The authors define RI by referring to an authoritative source, i.e. ENERI, in terms of ‘attitude and habit of researchers.' Defining RI in terms of personal characteristics of the researchers (i.e. their attitudes and habits) is a legitimate choice, but it does not cover all the meanings of RI that we find in the literature. In addition to RI as ‘integrity of researchers’, there are at least two other meanings: integrity of the research (which can be defined in a narrow and broad sense encompassing all ethical issues in research), and integrity of the institutions and systems (Helgesson and Bülow, 2021). Far from being a mere philosophical issue, Helgesson and Bülow argue that confusion of these different meanings can lead to value conflicts.

 I am not suggesting that the authors offer a fully-fledged philosophical defence of the choice of RI as ‘integrity of researchers’, but I recommend they acknowledge this plurality of meanings and motivate their choice in the context of their study (e.g. it is instrumental to their aim to study researchers’ perspectives), and clarify whether this was the meaning of RI included in the RI initiatives the participants were exposed to, or whether the meaning offered to the participants somehow fluctuated between different interpretations. In addition, considering different meanings of RI can add depth to the discussion of the results: do the participants tend to interpret RI as personal characteristics (habits and attitudes) or do other meanings emerge? Do these different meanings stem from the researchers’ own reflections or were they included in the training material and/or in the discussions? Staying within an interpretation of RI as ‘integrity of researchers’, the authors might find useful the work of Vincent and Hooper (2025) on thick and thin RI. For example, PhD student 3 complaining that the principles of RI are “fairly abstract terms” could be explained by codes of conduct for RI displaying, necessarily, a thin ethics. Engaging with these sources or similar ones will make the discussion section more robust.

***Is the study design appropriate and is the work technically sound?***

 Partially. The methods employed are appropriate to the research questions, and the authors make the very appreciated effort to describe their positionality in a reflexive way. However, there are two points that need to be clarified.

 First, the two authors have independently analysed the essays. Differences in the codes were used “as a source of insight for a series of reflexive discussions on the initial codes and preliminary themes and how these were influenced by each researcher’s relationship with the context and the research subject”. I think this is a methodological highlight of this work, something that makes it stand out among too many empirical works claiming to use reflexive TA but then pursuing consensus among coders. However, some clarification is needed about the authors’ joint reflexive discussions to distinguish them more clearly from a form of consensus-reaching: were there cases where disagreement persisted, or did the authors reach agreement on each of the cases? How did the authors’ joint reflexive discussion take place in practice? Did the authors develop themes together starting from possibly different codes?

 Second, in the section ‘Strengths, limitations and future research’, the authors talk about ‘thematic saturation’. Saturation is quite a contested concept in the context of TA: “The concept of data saturation – widely touted as a ‘gold standard’ for determining ‘sample size’ – is also deeply problematic (Braun & Clarke, 2019b). We recommend avoiding claims of ‘saturation’; instead, we find concepts such as information power useful (Malterud, Siersma, & Guassora, 2016).” (Braun and Clarke, 2022, p. 71). Following the suggestion by Braun and Clarke, the authors might want to check out whether ‘information power’ is more useful for their aims.

**Minor point**

 In the Introduction, the authors compare RI to meta-research, which encompasses all ‘research on research’. I find it odd to compare RI as ‘the attitude and habit of the researchers’, which could be a research topic, to a research field (research on research). I guess the authors wanted to compare *research* on RI with research on research, i.e. meta-research.

If applicable, is the statistical analysis and its interpretation appropriate?

Not applicable

Is the study design appropriate and is the work technically sound?

Partly

Is the work clearly and accurately presented and does it engage with the current literature?

Partly

Are the conclusions drawn adequately supported by the results?

Yes

Are sufficient details of methods and analysis provided to allow replication by others?

Not applicable

Are all the source data and materials underlying the results available?

No source data required

Reviewer Expertise:

Philosophy of science, research integrity, scholarly communication, qualitative research

I confirm that I have read this submission and believe that I have an appropriate level of expertise to confirm that it is of an acceptable scientific standard, however I have significant reservations, as outlined above.

## References

[1] : Research Integrity and Hidden Value Conflicts. *Journal of Academic Ethics* .2023;21(1) : 10.1007/s10805-021-09442-0113-123 10.1007/s10805-021-09442-0

[2] : Research integrity, thick and thin. *Synthese* .2025;206(2) : 10.1007/s11229-025-05157-6 10.1007/s11229-025-05157-6

[3] : Sample Size in Qualitative Interview Studies. *Qualitative Health Research* .2016;26(13) : 10.1177/10497323156174441753-1760 10.1177/1049732315617444 26613970

# Reviewer report: Reviewer response for version 1

**DOI:** 10.21956/openreseurope.25601.r77285

**Published:** 2026-08-07

**Authors:** Sadia Yaseen

**Affiliations:** 1 Rashid Latif Medical College Lahore, Lahore, Pakistan

**Overall Methodological Presentation**

 The manuscript is clearly presented and provides good methodological transparency, particularly regarding participant recruitment, positionality, reflexive analysis, and the use of quotations. However, some areas require clarification, particularly participant terminology, data collection procedures, essay length, and the alignment of the analytical approach with reflexive thematic analysis.

**Participant Terminology**

 The term **“participating researchers”** is confusing. The authors should consider using **“participants”** instead, as it is already stated in the inclusion criteria that all participants were researchers, whether PhD candidates, postdoctoral researchers, or interns.

**Participant Approach and Submission of Reflections**

 It is not mentioned how the participants were approached or how they submitted their reflective essays. Please provide clarification regarding the method used to invite participants and the process through which the reflections were submitted.

**Timing of Data Collection**

 The manuscript states that data were collected halfway through the project. It would be better to specifically mention the **month and year** in which the reflective essays were collected to provide greater clarity regarding the timing of data collection.

**Variation in Essay Length**

 The length of the reflective essays varies considerably. The longest essay is approximately four times longer than the shortest essay. This variation should be acknowledged in the manuscript, as differences in the length of reflections may potentially influence the depth and richness of the data.

**Description of Essays as “Very Rich Texts”**

 The authors describe the essays as **“very rich texts,”** but this assertion requires support from the data rather than from the intention of the prompt. Please clarify whether the subsequent guidance regarding essay length was provided uniformly to all participants and discuss whether the change in instructions may have influenced the depth or length of the responses.

**Reflexive Thematic Analysis**

 The analysis process is described in detail; however, I have some concern regarding its alignment with reflexive thematic analysis. The authors describe independent coding, comparison of coding, discussion of discrepancies, development of a single coding scheme, and achieving greater consistency in coding. This appears somewhat reliability oriented. Please clarify how this process is consistent with the principles of reflexive thematic analysis.

**Alignment of Methodology With the Study Rationale**

 The authors should further clarify the reflexive thematic analysis approach and strengthen the alignment between the **domain-specific rationale** of the study and the findings. In particular, the limited reflection by participants on domain-specific RI challenges should be more critically addressed, given that domain specificity is central to the study rationale.

**Interpretation of Findings**

 The discussion and conclusion occasionally appear to overstate the findings. As the study explored participants’ reflective accounts, claims regarding improved research practice, the value of communal reflection over formal courses, and the ability to **“properly act”** upon RI dilemmas should be framed as perceived benefits or intentions rather than demonstrated outcomes. In addition, the limited reflection on domain-specific RI challenges deserves more critical discussion, given that domain specificity is central to the study rationale.

If applicable, is the statistical analysis and its interpretation appropriate?

Not applicable

Is the study design appropriate and is the work technically sound?

Yes

Is the work clearly and accurately presented and does it engage with the current literature?

Yes

Are the conclusions drawn adequately supported by the results?

Yes

Are sufficient details of methods and analysis provided to allow replication by others?

Partly

Are all the source data and materials underlying the results available?

Yes

Reviewer Expertise:

Qualifications: MBBS, MPhil (Histopathology), MHPE, PGD in Medical Journalism.Research Areas: Medical and health professions education, curriculum and assessment, qualitative research, faculty development, educational innovation, interprofessional education, research methodology, medical journalism, and research integrity.

I confirm that I have read this submission and believe that I have an appropriate level of expertise to confirm that it is of an acceptable scientific standard, however I have significant reservations, as outlined above.
